# Cytoplasmic Sulfurtransferases in the Purple Sulfur Bacterium *Allochromatium vinosum:* Evidence for Sulfur Transfer from DsrEFH to DsrC

**DOI:** 10.1371/journal.pone.0040785

**Published:** 2012-07-16

**Authors:** Yvonne Stockdreher, Sofia S. Venceslau, Michaele Josten, Hans-Georg Sahl, Inês A. C. Pereira, Christiane Dahl

**Affiliations:** 1 Institut für Mikrobiologie and Biotechnologie, Rheinische Friedrich-Wilhelms-Universität Bonn, Bonn, Germany; 2 Instituto de Tecnologia Química e Biológica, Universidade Nova de Lisboa, Avenida da República, Oeiras, Portugal; 3 Institut für Medizinische Mikrobiologie, Immunologie and Parasitologie, Abteilung Pharmazeutische Mikrobiologie, Rheinische Friedrich-Wilhelms-Universität Bonn, Bonn, Germany; Louisiana State University and A & M College, United States of America

## Abstract

While the importance of sulfur transfer reactions is well established for a number of biosynthetic pathways, evidence has only started to emerge that sulfurtransferases may also be major players in sulfur-based microbial energy metabolism. Among the first organisms studied in this regard is the phototrophic purple sulfur bacterium *Allochromatium vinosum*. During the oxidation of reduced sulfur species to sulfate this Gammaproteobacterium accumulates sulfur globules. Low molecular weight organic persulfides have been proposed as carrier molecules transferring sulfur from the periplasmic sulfur globules into the cytoplasm where it is further oxidized via the “Dsr” (**d**issimilatory **s**ulfite **r**eductase) proteins. We have suggested earlier that the heterohexameric protein DsrEFH is the direct or indirect acceptor for persulfidic sulfur imported into the cytoplasm. This proposal originated from the structural similarity of DsrEFH with the established sulfurtransferase TusBCD from *E. coli*. As part of a system for tRNA modification TusBCD transfers sulfur to TusE, a homolog of another crucial component of the *A. vinosum* Dsr system, namely DsrC. Here we show that neither DsrEFH nor DsrC have the ability to mobilize sulfane sulfur directly from low molecular weight thiols like thiosulfate or glutathione persulfide. However, we demonstrate that DsrEFH binds sulfur specifically to the conserved cysteine residue DsrE-Cys78 *in vitro*. Sulfur atoms bound to cysteines in DsrH and DsrF were not detected. DsrC was exclusively persulfurated at DsrC-Cys111 in the penultimate position of the protein. Most importantly, we show that persulfurated DsrEFH indeed serves as an effective sulfur donor for DsrC *in vitro.* The active site cysteines Cys78 of DsrE and Cys20 of DsrH furthermore proved to be essential for sulfur oxidation *in vivo* supporting the notion that DsrEFH and DsrC are part of a sulfur relay system that transfers sulfur from a persulfurated carrier molecule to the dissimilatory sulfite reductase DsrAB.

## Introduction

The chemically versatile persulfide group (RS-SH) participates in a wide array of biochemical pathways. In recent years, persulfurated proteins have not only been shown to supply a number of important and elaborate biosynthetic pathways with “activated sulfur” [1], [2] but there is accumulating evidence that the enzymatic generation of persulfidic sulfur and transfer of sulfane sulfur as persulfide is also an essential and so-far largely neglected component of dissimilatory sulfur oxidation pathways [3]. Even those reactions that until now have been considered to use sulfide as the immediate substrate likely require protein-bound persulfidic sulfur. A prominent example is the enzyme reverse dissimilatory sulfite reductase (DsrAB), that has long been suggested to catalyze the oxidation of free sulfide to sulfite in the course of the composite Dsr-involving pathway [4], [5]. This pathway involves the accumulation of sulfur globules as a transient product and occurs in many environmentally important photo- and chemolithoautotrophic bacteria [4], [6]. DsrAB is also present in sulfate-reducing bacteria where it is a major player in the reduction of sulfite to sulfide [7]. Structural and biochemical characterization of Dsr proteins from the phototrophic sulfur oxidizer *Allochromatium vinosum* [3], [8] and structural analysis of DsrAB from sulfate-reducing bacteria [9], [10], [11] now indicate a protein-bound persulfide instead of free sulfide as the immediate product/substrate of dissimilatory sulfite reductases.

Recent analysis of the sulfur oxidation pathway in *A. vinosum* revealed major similarities between the *E. coli* Tus sulfur relay system for tRNA modification and the Dsr proteins, which are evidently essential for sulfur oxidation [12], [13], [14]. The Tus proteins (TusA, TusBCD and TusE) in *E. coli* are sulfurtransferases involved in the biosynthesis of 2-thiouridine. They mediate the sulfur transfer between the cysteine desulfurase IscS and MnmA, a dedicated 2-thiouridylase [15]. Sulfur is derived from L-cysteine by IscS, which transfers it to the TusA. From here it is successively transferred to TusBCD and TusE. The latter interacts directly with MnmA. MnmA finally performs the modification of the tRNA. The TusBCD and TusE proteins show a high degree of similarity to the DsrEFH and DsrC proteins that reside in the cytoplasm of *A. vinosum*.

DsrEFH from *A. vinosum* is a hexameric protein arranged in a α_2_β_2_γ_2_ structure and harbours two conserved cysteine residues in the putative active sites of DsrE and DsrH. These residues are DsrE-Cys78 and DsrH-Cys20, respectively [3]. A *dsrE*-deficient mutant strain of *A. vinosum* was unable to degrade sulfur globules, indicating a crucial role for this protein in sulfur oxidation [3]. DsrEFH is not found in sulfate-reducing organisms, while DsrC occurs in sulfate reducers and sulfur oxidizers alike [13]. The eminently important role of this protein is underlined by recent metatranscriptome and metagenome analyses of environmental samples that identified *dsrC* to be the most abundant gene in communities of sulfur oxidizers and sulfate reducers [16], [17]. The active region of DsrC is its flexible carboxy-terminus. This region extends from the globular protein and harbours two highly conserved cysteine residues: DsrC-Cys100 and DsrC-Cys111 [8]. While the cysteine residue in the penultimate position of DsrC-Cys111 is strictly conserved in all DsrC sequences, the preceding cysteine is only found in organisms containing DsrAB as well. In those organisms lacking DsrAB but containing proteins of the TusE/DsrC/DsvC family (TIGR03342), the cysteine equivalent to DsrC-Cys100 is almost always replaced either by alanine, serine or threonine. Exceptions are found among the family *Methylococcaceae* and the genera *Marinobacter* and *Lawsonia*. In the latter DsrC/TusE homologs both conserved cysteine residues are present.

Previously, we demonstrated that DsrEFH and DsrC interact and this interaction is strictly dependent on the cysteine residues DsrC-Cys111 and DsrE-Cys78 [8]. In our attempt to further elucidate the mechanism of sulfur oxidation in *A. vinosum* we now provide experimental evidence that DsrEFH and DsrC act as a sulfurtransferase and a substrate-binding protein, respectively and thereby confirm our previous proposal concerning their function. Our experiments focus on the role of the conserved cysteine residues hosted by these proteins. Furthermore, we provide deeper insight into the interaction between DsrEFH and DsrC.

## Results

### The Cysteine Residues Cys78 of DsrE and Cys20 of DsrH are Essential for Sulfur Oxidation in *A. vinosum in vivo*

Deletion of the *dsrE* gene proved DsrEFH to be crucial for sulfur oxidation in *A. vinosum* since the Δ*dsrE* mutant was unable to oxidize sulfur globules stored as an intermediate during the oxidation of sulfide or thiosulfate [3]. The wild type phenotype could be restored by complementation *in trans* with the *dsrEFH* genes [3]. To gain a more detailed view on the protein’s mode of action and the relevance of the conserved cysteine residues in DsrE and DsrH, we complemented the *A. vinosum* Δ*dsrE* mutant strain with *dsrEFH* sequences carrying Cys/Ser exchanges at positions DsrE-Cys78 and/or DsrH-Cys20.

**Figure 1:**
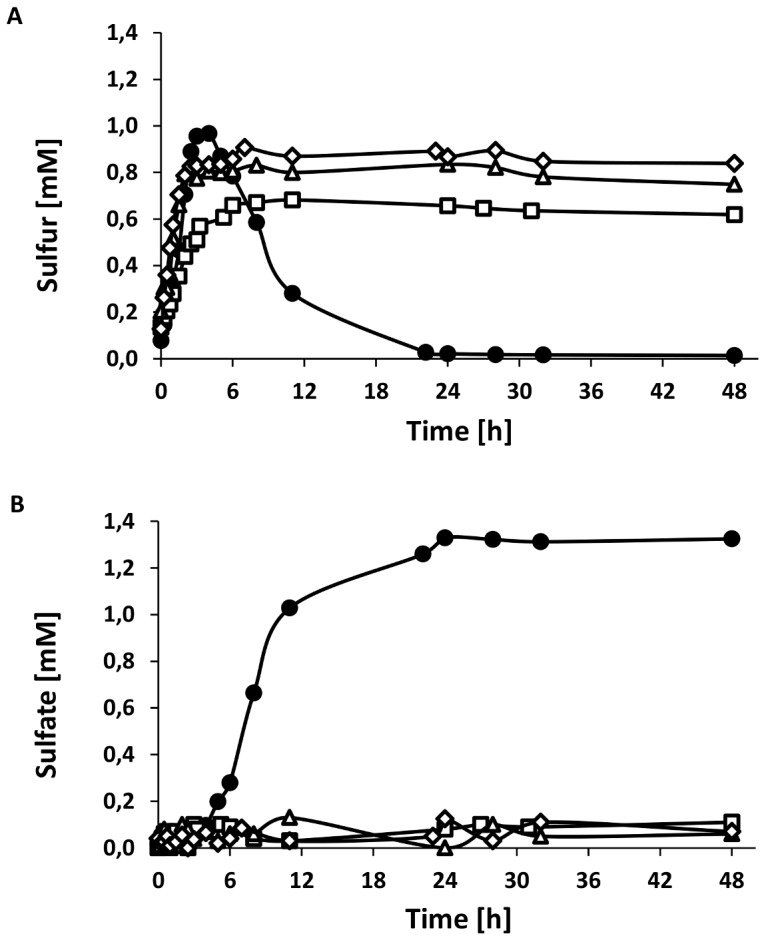
Accumulation of sulfur and formation of sulfate by different *A. vinosum* strains. Panel A shows accumulation of sulfur, in panel B formation of sulfate is depicted. Cells of *A. vinosum* wild type (•), *A. vinosum* Δ*dsrE+dsrE_78_FH* (□), *A. vinosum* Δ*dsrE*+*dsrEFH_20_* (Δ) and *A. vinosum* Δ*dsrE*+*dsrE_78_FH_20_* (◊) were grown photolithoautotrophically with 2 mM sulfide as sole electron source. Discrepancy of sulfate and initially supplied sulfide is due to loss of gaseous sulfide. Protein concentrations increased from approximately 90 to 140 µg/ml in all cultures.

The cultures were grown photolithoautotrophically with 2 mM sulfide as the sole electron source. The wild type control cultures showed a phenotype as expected: sulfide was rapidly converted to sulfur and transiently accumulated in sulfur globules. The sulfur was completely oxidized to the final product sulfate within 24 hours. In contrast, none of the complementation mutants, *A. vinosum* Δ*dsrE* + *dsrE_C78S_FH*, *A. vinosum* Δ*dsrE*+ *dsrEFH_C20S_* or *A. vinosum* Δ*dsrE* + *dsrE_C78S_FH_C20S_* was able to degrade the sulfur globules (Fig. 1A). Although sulfide was oxidized to sulfur at rates similar to the wild type, stored sulfur could not be further metabolized. Consistent with this finding, sulfate was not detected (Fig. 1B). These results provide clear evidence that both cysteine residues, DsrE-Cys78 and DsrH-Cys20, have a crucial role for sulfur oxidation in *A. vinosum* and support the assumption that DsrEFH acts as a sulfurtransferase.

### DsrEFH and DsrC Form a Stable Protein Complex

**Figure 2:**
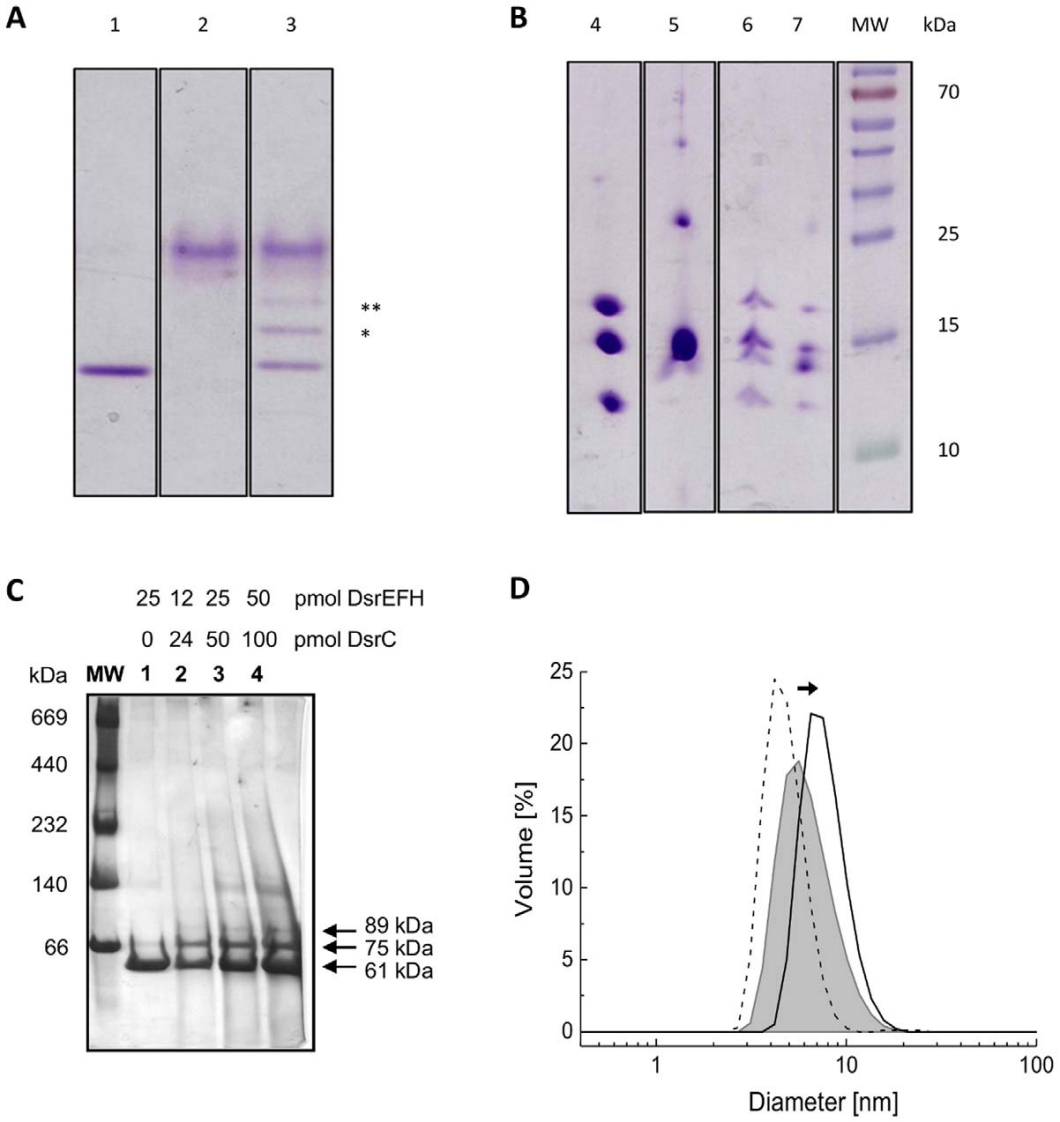
Formation of stable protein complexes between DsrC and DsrEFH analysed with electrophoretic methods. (**A**) For the interaction, 200 pmol of DsrEFH and 400 pmol of DsrC were incubated for 30 minutes at 30°C. The protein mixtures were then applied to a native polyacrylamide gel (7.5%). The additional bands that indicate DsrEFH/DsrC complexes are marked by * and **. All bands were cut out of the gel and the pieces were applied to SDS-PAGE (15%). (**A**) Proteins in native gel (lanes 1–3): lane 1 DsrEFH, lane 2 DsrC, lane 3 DsrEFH pre-incubated with DsrC; (**B**) SDS-PAGE (lanes 4–7): lane 4 DsrEFH, lane 5 DsrC, lane 6 DsrEFH and DsrC (lower migrating band), lane 7 DsrEFH and DsrC (upper migrating band). Molecular weight (MW) of marker proteins is given in kDa. (**C**) Proteins in Blue-native PAGE (10–15%): lane 1 DsrEFH, lane 2 to 4 DsrEFH pre-incubated with DsrC in 1∶2 ratio but with increasing amounts. (**D**) DLS measurements of DsrEFH (black line) and DsrC (dashed line) in solution, and when both proteins were pre-incubated together at 30°C (grey line).

The interaction between DsrEFH and DsrC *in vitro* and its dependency on the residues DsrE-Cys78 and DsrH-Cys20 have been first demonstrated by band shift assays in native polyacrylamide gels [8]. The migration patterns of DsrC and DsrEFH in native polyacrylamide gels changed after both proteins were incubated together: two additional bands appeared between DsrC and DsrEFH (Fig. 2A). So far it was not shown unambiguously that these additional bands indeed arose by formation of complexes between DsrEFH and DsrC. Therefore, we extracted these bands from the native gel, applied them to SDS-PAGE and visualized the proteins engaged in the formation of the additional bands. As shown in Fig. 2B both bands contained DsrEFH as well as DsrC. Notably, the signal for DsrC was significantly stronger in the upper than in the lower band, indicating that the protein complex in the upper band (Fig. 2B, lane 7) had a higher DsrC:DsrEFH ratio. We suggest the presence of two DsrC per DsrE_2_F_2_H_2_ for the complex migrating as the upper band and the presence of only one DsrC per DsrE_2_F_2_H_2_ heterohexamer for the faster migrating complex.

This was confirmed by Blue-native gel analysis, which allows separation of native proteins based only on molecular mass. Two extra bands were also observed in the blue-native gel when DsrEFH was incubated with DsrC (Fig. 2C, lane 4). Using a calibration curve based on the molecular masses of the standards, those bands correspond to an extra 14 and 28 kDa molecular mass, respectively, compared with DsrEFH alone. These additional masses agree with one and two molecules of DsrC per DsrE2F2H2 heterohexamer, respectively, with the most intense band corresponding to the DsrC:DsrEFH complex in a 1∶1 ratio. DsrC runs as a smear in blue-native PAGE and it is not visualized in this gel.

Dynamic light scattering (DLS) was also used to get a picture of the population size upon *in vitro* formation of the DsrC:DsrEFH complex by following changes in the DsrEFH hydrodynamic diameter upon incubation with DsrC. DLS analysis of the DsrC:DsrEFH mixture indicates the presence of a particle with a hydrodynamic diameter (7.8 nm) that is 22% higher than that of DsrEFH alone (6.4 nm) (Fig. 2D), thereby supporting the formation of a complex with DsrC (4.8 nm). The apparent molecular mass of the complex determined by this technique corresponds to a 1∶1 complex stoichiometry. Nevertheless, it is important to note that the band centered around 7.8 nm diameter is quite broad, so less abundant complexes with other stoichiometries may be present, which cannot be resolved by this technique.

**Figure 3:**
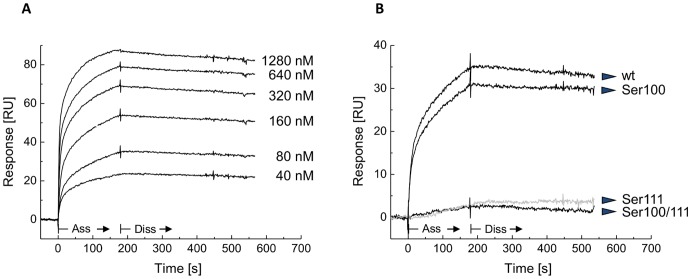
Analysis of the protein-protein interaction between DsrEFH and DsrC by Surface Plasmon Resonance. (**A**) and (**B**) are sensorgrams of interaction of DsrC (wt and cysteine mutated) with DsrEFH determined by surface plasmon resonance affinity assays. Various concentrations (ranging from 40 to 1280 nM) of DsrC wild type were injected through the flow cell with immobilized DsrEFH. The kinetic profiles are shown in (A). Comparison of binding levels using different reduced analytes (80 nM): DsrC wild type, DsrC-Ser100, DsrC-Ser111 and double cysteine mutated DsrC (B). Ass, association step; Diss, dissociation step.

To further characterize the interaction between DsrEFH and DsrC and evaluate the role of the two conserved DsrC cysteine residues in the formation of the complex we used Surface Plasmon Resonance (SPR), which allows dynamic studies of the complex association and dissociation. DsrEFH was immobilized on the SPR sensor chip and its binding to the DsrC wild type protein took place in a concentration-dependent manner (Fig. 3A). A higher level of response was observed when the cysteines were previously reduced (data not shown). The binding curve showed a fast on-rate. Practically no dissociation was observed, indicating that the complex is very strong, as previously indicated by the native gel technique. The association and dissociation rate constants could not be derived from the SPR experiments since no kinetic model could fit the experimental data satisfactorily. Indeed, the resulting interaction curves lead to complex responses most probably due DsrE_2_F_2_H_2_ interacting with either one or two molecules of DsrC, in agreement with the results obtained after native PAGE. To study the relevance of the conserved cysteine residues at the DsrC carboxy-terminus in the interaction with DsrEFH, we conducted SPR experiments at the same analyte concentration using DsrC-Ser100 and DsrC-Ser111 single mutants and a DsrC-Ser100/111 double mutant (Fig. 3B). A strongly decreased binding was observed for DsrC-Ser111 and DsrC-Ser100/111 when compared to the wild type or DsrC-Ser100, which behaved similarly. This is consistent with previously reported data that showed that DsrEFH interacts with DsrC via Cys111 rather than Cys100 [8]. Similar results were obtained for other analyte concentrations.

### DsrEFH and DsrC Bind Sulfur

In a first step to assess the possibility that DsrEFH and DsrC may act as sulfurtransferases, their sulfur binding abilities were tested. For this purpose both proteins were incubated either with the *E. coli* cysteine desulfurase IscS and cysteine or with sulfide alone. Afterwards, the proteins were analysed via MALDI-TOF mass spectrometry. An additional mass of 32 Da compared to the proteins’ genuine mass is characteristic for persulfuration. Note that for interpretation of the spectra singly charged molecules as well as doubly charged molecules were used. Furthermore, the exchange of cysteine with serine in mutated proteins causes a 16 Da reduction of their molecular masses.

**Figure 4:**
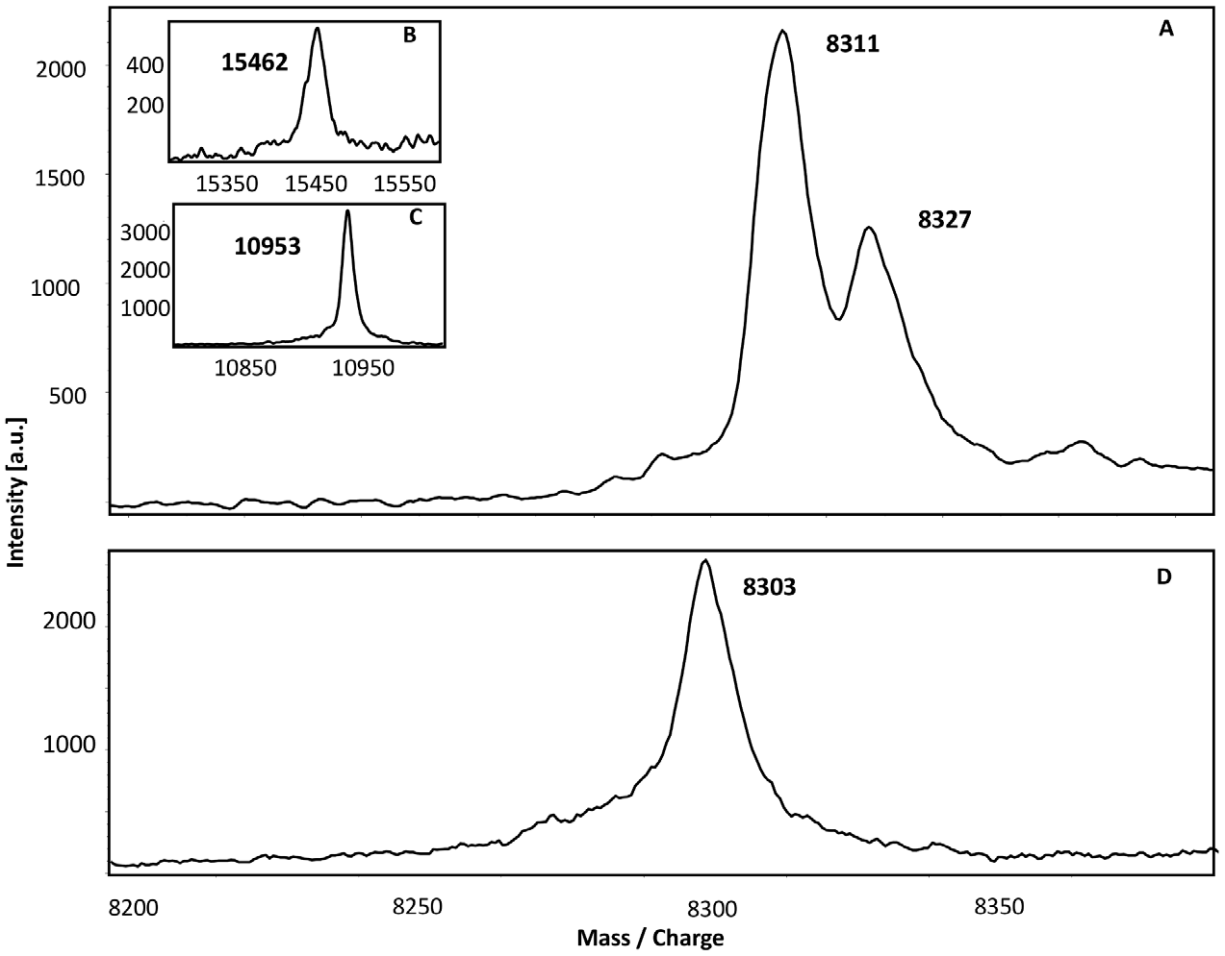
MALDI-TOF spectra of persulfurated DsrEFH proteins. 30 µM of unmodified DsrEFH and protein carrying a Cys-Ser mutation in DsrE were incubated with 2 µM IscS and 2 mM cysteine or 2 mM sulfide. Binding of sulfur atoms is indicated by an additional mass of 32 Da for singly charged molecules and 16 Da for double charged molecules. Note that results are shown for double charged proteins. **(A)** DsrE.(**B**) DsrF. (**C**) DsrH. (**D**) DsrE_78_.

During mass spectrometry the protein DsrEFH decomposed into its subunits, so each subunit could be analysed individually. For each protein a mass could be detected that agreed with the calculated mass within a range of max. 2 Da. After incubation with a sulfur donor the spectrum of doubly charged DsrE (Fig. 4) showed two species of the protein: the first species represented the mass of recombinant DsrE. With an additional mass of 16 Da as expected for doubly charged molecules the second species displayed the persulfurated version of DsrE. The persulfuration was attained by incubation with sulfide as well as with cysteine and IscS. There was no additional mass detected when the mutated protein containing DsrE-Ser78 was incubated with a sulfur donor (Fig. 4D). Interestingly, neither DsrH nor DsrF were able to bind sulfur atoms though both polypeptides carry cysteine residues. Since the cysteine residue in DsrH is essential for sulfur oxidation it is noteworthy that the exchange of this residue had no effect on the sulfur binding ability of DsrE.

**Figure 5:**
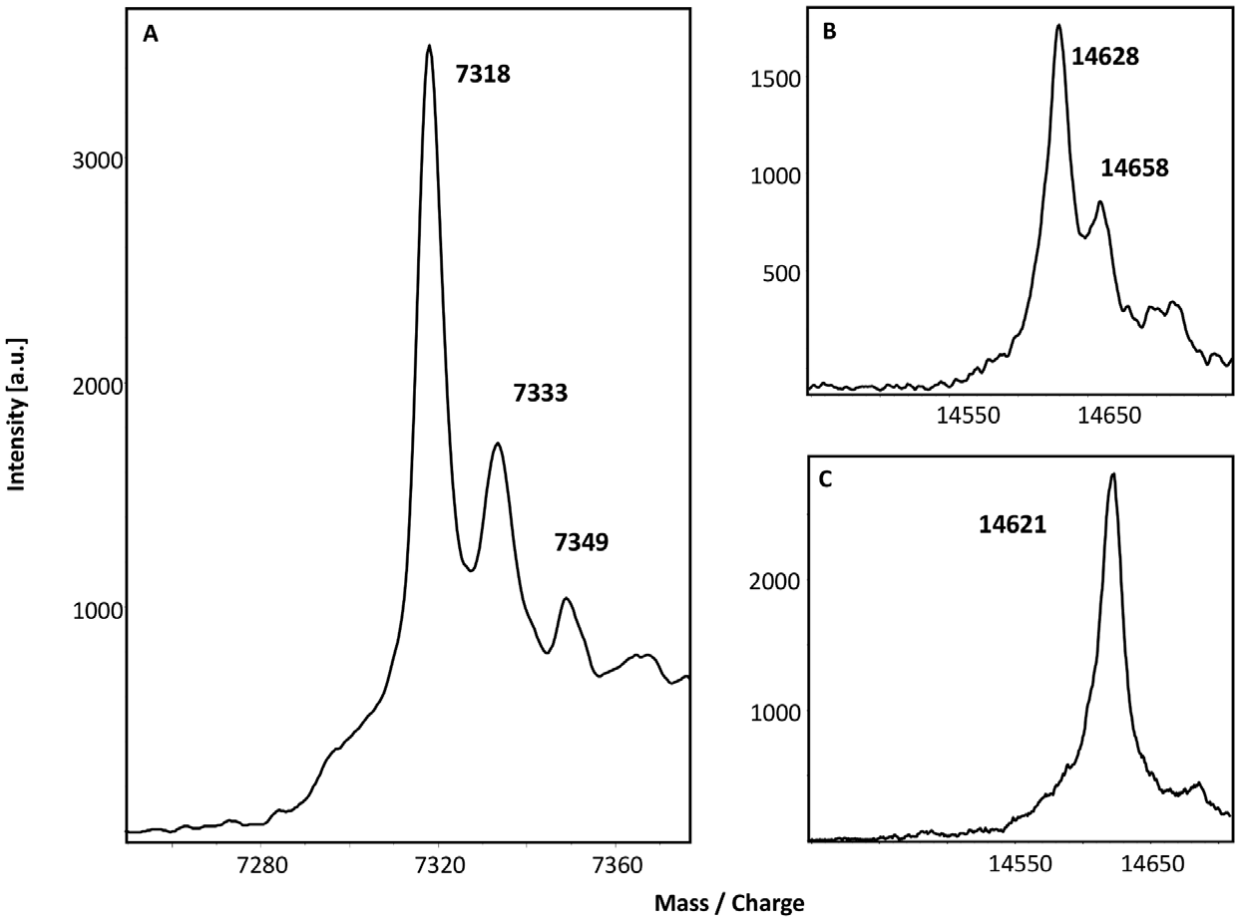
MALDI-TOF spectra of persulfurated DsrC proteins. 30 µM of unmodified DsrC **(A)** and DsrC mutant proteins carrying a Cys-Ser mutation in DsrC-Cys100 (**B**) or DsrC-Cys111 (**C**) were incubated with 2 µM IscS and 2 mM cysteine or with 2 mM sulfide. Note that for the unmodified DsrC results are shown for the double charged molecule.

The spectrum for doubly charged DsrC is shown in Fig. 5. The first peak matches the theoretically calculated molecular mass within a tolerance range of 1 Da. The second peak exhibited a mass increase of 16 Da, which is characteristic for the persulfidic state of the protein. Unlike DsrEFH, DsrC was able to bind even a second sulfur atom. To clarify the question whether sulfur atoms were specifically bound to one of the carboxy-terminal conserved cysteines of DsrC, sulfur binding experiments were also performed with mutated DsrC proteins. Additional peaks were still observed when the DsrC protein with a Cys/Ser exchange in DsrC-Cys100 was tested, however, sulfur binding capability was obviously lost when DsrC-Cys111 was mutated.

Sulfite was tested as a further possible substrate for DsrC. Sulfite reacts with proteins by reducing existing inter- or intramolecular disulfide bonds according to the equation 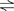 [18]. Besides the peak for unaltered DsrC, a second peak with molecular mass increased by 80 Da occurred in the spectrum (Figure S1). The second peak represented a DsrC molecule with a sulfonate group bound. Repeating this experiment with mutated DsrC proteins demonstrated that DsrC-Cys111 is the residue which is responsible binding the sulfonate group. Together, our findings provide evidence that the capability of binding sulfur species is restricted exclusively to the Cys111 at the penultimate position of DsrC whereas DsrC-Cys100 is irrelevant for these reactions.

### Neither DsrEFH nor DsrC React with Thiosulfate or Glutathione Persulfide as Sulfur Donors

Persulfidic glutathione (GSSH) and gluthatione amide (GASH) are discussed as candidates for transferring sulfur from the periplasmic sulfur globules to the cytoplasm [19], [20]. However, neither DsrEFH nor DsrC were able to use GSSH or thiosulfate as substrates. MALDI-TOF spectra obtained after incubation of the proteins with these substrates revealed that sulfur was not bound to the proteins. This indicates that none of the two proteins is able to mobilize sulfane sulfur from GSSH or thiosulfate. In agreement, both proteins did not show any activity as thiosulfate:cyanide sulfurtransferase (rhodanese) or glutathione persulfide:cyanide sulfurtransferase. Thiosulfate reductase activity with dithiotreitol as electron donor was also neither detected for DsrEFH nor for DsrC.

### DsrEFH Transfers Sulfur to DsrC

In the next step, experiments were carried out in order to study the sulfur transfer capabilities of DsrEFH and DsrC. For these studies, sulfide was always used as the agent for protein persulfuration in order to exclude involvement of IscS in the following reactions. Thus it was guaranteed that a successful persulfuration of the respective acceptor protein could be traced back exclusively to the activity of the donor protein, DsrEFH or DsrC. To absolutely rule out unspecific persulfuration of the acceptor protein by residual sulfide added initially to the donor protein, sulfide was quantitatively removed from the donor protein via PD Mini–Trap columns (GE Healthcare, Munich, Germany).

**Figure 6:**
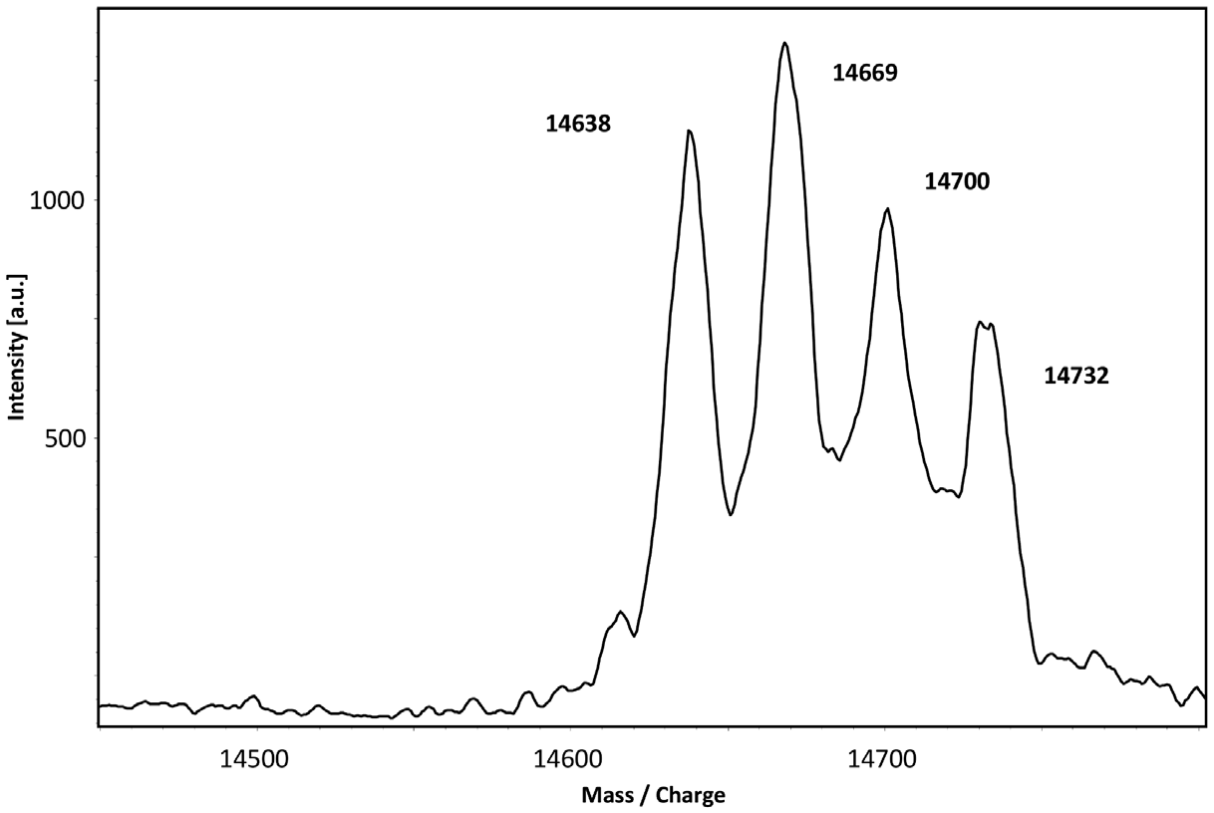
MALDI-TOF spectrum of DsrC. 30 µM DsrC was incubated with 30 µM persulfurated DsrEFH for 1 hour at 30°C. The transfer of up to three sulfur atoms from DsrEFH to DsrC is documented by mass increase in steps of 32 Da.

In a first experiment, the ability of DsrEFH to serve as sulfur donor for DsrC was tested. DsrEFH was incubated with sulfide and sulfur was successfully bound to DsrE-Cys78 as shown by mass spectrometry. After removal of sulfide, persulfurated DsrEFH was incubated with DsrC. Mass spectrometric analysis showed appearance of three new signals corresponding to higher masses in addition to native DsrC (Fig. 6). Each of these three extra peaks showed a mass increase of 31 or 32 Da compared to the previous peak and therefore each represented one sulfur atom bound to DsrC. In the next step, mutated DsrC proteins were tested for their suitability as sulfur acceptors. In accordance with the results for the sulfur binding experiments, sulfane sulfur could only be transferred from persulfurated DsrEFH to those DsrC variants that still contained cysteine residue Cys111. We conclude that DsrEFH does in fact effectively transfer sulfur to DsrC *in vitro* and thereby establishes a short polysulfide chain on DsrC-Cys111.

As shown above, the conserved cysteine in DsrH, DsrH-Cys20, itself was not able to bind sulfur nor was it required for the sulfur binding by DsrE. In order to test a possible involvement of DsrH-Cys20 in the sulfurtransferase activity of the DsrEFH, the wild type protein was replaced by a protein carrying a serine in position 20 of DsrH. The results were the same as shown for the wild type DsrEFH: Again three sulfur atoms were transferred to DsrC. This clearly demonstrates that DsrH-Cys20 is not essential for the sulfurtransferase activity of DsrEFH *in vitro*. Sulfur transfer from DsrEFH to DsrC was only prevented in the absence of DsrC-Cys111.

In a further series of experiments, the ability of DsrC to donate sulfur to DsrEFH was assessed. To this end, DsrC was persulfurated with sulfide, the sulfur compound was completely removed and persulfurated DsrC was afterwards incubated with DsrEFH and its three different Cys/Ser variants. However, sulfur transfer from DsrC to DsrEFH was not observed in any of theses cases.

In summary, DsrEFH can effectively pass on sulfane sulfur to DsrC *in vitro*. More precisely, the transfer was accomplished from DsrE-Cys78 to DsrC-Cys111 without a requirement for DsrH-Cys20 and DsrC-Cys100. Sulfur transfer from DsrC to DsrEFH was not detected.

## Discussion

DsrEFH plays an essential role in the oxidation of stored sulfur globules in *A. vinosum* [3]. The data presented in this work demonstrate the significance of the conserved cysteines DsrE-Cys78 and DsrH-Cys20 for an efficiently working Dsr system *in vivo*. Mutation of both residues led to a phenotype that differed from that of the wild type phenotype as well as from the *A. vinosum* Δ*dsrE*+*dsrEFH* complementation mutant [3]. While these latter strains degraded stored sulfur globules within 24 hours and produced sulfate as the end product, the mutants presented here were neither able to oxidize sulfur globules nor to produce sulfate.

Our former report on the dependency of the interaction between DsrEFH and DsrC *in vitro* on cysteine 78 of DsrE [3], together with the critical role of the corresponding residue TusD-Cys78 of *E. coli* TusBCD for sulfurtransferase activity [15], led to the assumption that DsrE-Cys78 would be essential for the protein’s function *in vivo*. Indeed, our experiments proved this residue to be absolutely crucial. However, the fact that DsrH-Cys20 also fundamentally contributes to the oxidation of sulfur globules in *A. vinosum* was surprising.

Naturally, *in vitro* conditions do not fully reflect the environment in living cells. Persulfides are probably more favourable sulfur donors than sulfide for DsrEFH *in vivo*, since they provide a more controlled way for specific delivery of active sulfur to its target sites within living cells. The most significant difference between the conditions *in vivo* and *in vitro* is the actual sulfur donor for DsrEFH. While DsrEFH was incubated with sulfide for the experiments, in the cell a protein directly interacting with DsrEFH and thereby conducting the sulfur transfer from a low molecular weight organic persulfide to DsrEFH is more likely. It is possible that DsrH-Cys20 is essential for the interaction of DsrEFH with this as yet unknown sulfur donor.

The results presented here allow assigning a function to DsrEFH in *A. vinosum*. We show that DsrEFH is indeed a sulfurtransferase and that it conducts transfer of sulfur to DsrC *in vitro*. When incubated with sulfide, sulfur atoms are bound specifically to DsrE-Cys78. In the persulfurated state DsrEFH serves as sulfur donor for DsrC *in vitro*. The presence of the conserved Cys20 in DsrH is not required for the sulfur transfer.

This finding is in line with the observation for the corresponding TusBCD protein from *E. coli* [21]. Numata et al. [21] complemented *tusC* and *tusD* deficient *E. coli* mutants with *tusC* and *tusD* sequences that carried mutations of the cysteine residues in the putative active sites and analyzed the production of 2-thiouridine in the complementation strains. They found TusD-Cys78 to be the only essential cysteine residue for the sulfur transfer reaction. We therefore postulate that the cysteine residue in position 78 in DsrE/TusD is the active site for the DsrE superfamily (cl00672).

Our data revealed DsrC-Cys111 to be the sulfur binding site of DsrC. After incubation with sulfide or persulfidic DsrEFH, we detected up to three sulfur atoms bound to this residue. Mutation of DsrC-Cys111 resulted in the loss of sulfur binding ability of the protein and, concomitantly, had a substantial effect on the interaction with DsrEFH, as it was illustrated in SPR assays by a much weaker interaction. Notably, DsrC-Cys111 was also identified as the binding site for sulfite. The preceding cysteine DsrC-Cys100, which is strictly conserved in organisms with a sulfur-based energy metabolism [8], was dispensable for both reactions. We did not detect sulfur transfer from DsrC to DsrEFH, though the transfer would be possible in the opposite direction.

Besides the fact that DsrAB is copurified with DsrC and other Dsr proteins from *A. vinosum* in a supercomplex [22], only little is known about the interaction of DsrAB and DsrC in sulfur-oxidizing bacteria. Nevertheless, the structures of DsrAB from the sulfate reducers *Desulfovibrio vulgaris, D. gigas* and *Desulfomicrobium norvegicum* provide insights into this topic [9], [10], [11]. In these cases the DsrC proteins insert their C-terminal flexible arm into a cleft between DsrA and DsrB. In the three structures the main species present has a covalent bond between the catalytic siroheme and the terminal DsrC cysteine, which was proposed to result from an *in vitro* non-physiological reaction [9]. However, in the structure from *D. gigas* two other DsrC conformations are observed where this covalent bond is not present. In one of them the DsrC arm is extended and the terminal cysteine is found closer to a sulfite molecule bound in the substrate pocket. In the other conformation the flexible C-terminus is turned away from the siroheme and towards the other cysteine of DsrC. Oliveira et al. proposed a two-step mechanism for the sulfite reduction [9], where sulfite is considered to be reduced to a S^0^ intermediate consuming four electrons that are provided by an unknown donor. The sulfur atom is then transferred to the terminal DsrC cysteine (corresponding to *A. vinosum* DsrC-Cys111). Afterwards the flexible arm swings away from the catalytic site and comes in close proximity to the other DsrC cysteine (corresponding to *A. vinosum* DsrC-Cys100), which finally reduces the sulfur atom by forming a disulfide bond with DsrC-Cys104. The two electrons to restore the thiol-form of DsrC are thought to be delivered by the DsrMKJOP complex. Although in *A. vinosum* DsrAB is supposed to work in the reverse direction, i.e. oxidizing sulfur to sulfite, the general arrangement of DsrC and DsrAB in the complex should be similar to the proteins in sulfate-reducing bacteria. Here, we demonstrate that DsrC from *A. vinosum* accepts and binds sulfur that is delivered by DsrEFH to its penultimate cysteine. This finding strongly supports the proposal that DsrC serves as substrate-binding protein for DsrAB in sulfur oxidizers. In its persulfidic state DsrC could bring sulfane sulfur in contact with the catalytic siroheme of DsrAB where the sulfur is then oxidized. Afterwards DsrC might dissociate from DsrAB in a sulfonated form. Since DsrC can bind sulfite and this reaction is reversible the sulfonate group could be reductively released as sulfite by the formation of a disulfide bridge between the two cysteine residues of DsrC and then be further oxidized to sulfate. After the reduction of the disulfide bond DsrC can enter the cycle again. DsrK is a likely candidate for the reduction of DsrC [23]. This protein is related to the catalytic subunit of the heterodisulfide reductase HdrD in methanogenic archaea and has recently been shown to interact directly with DsrC [23].

**Figure 7:**
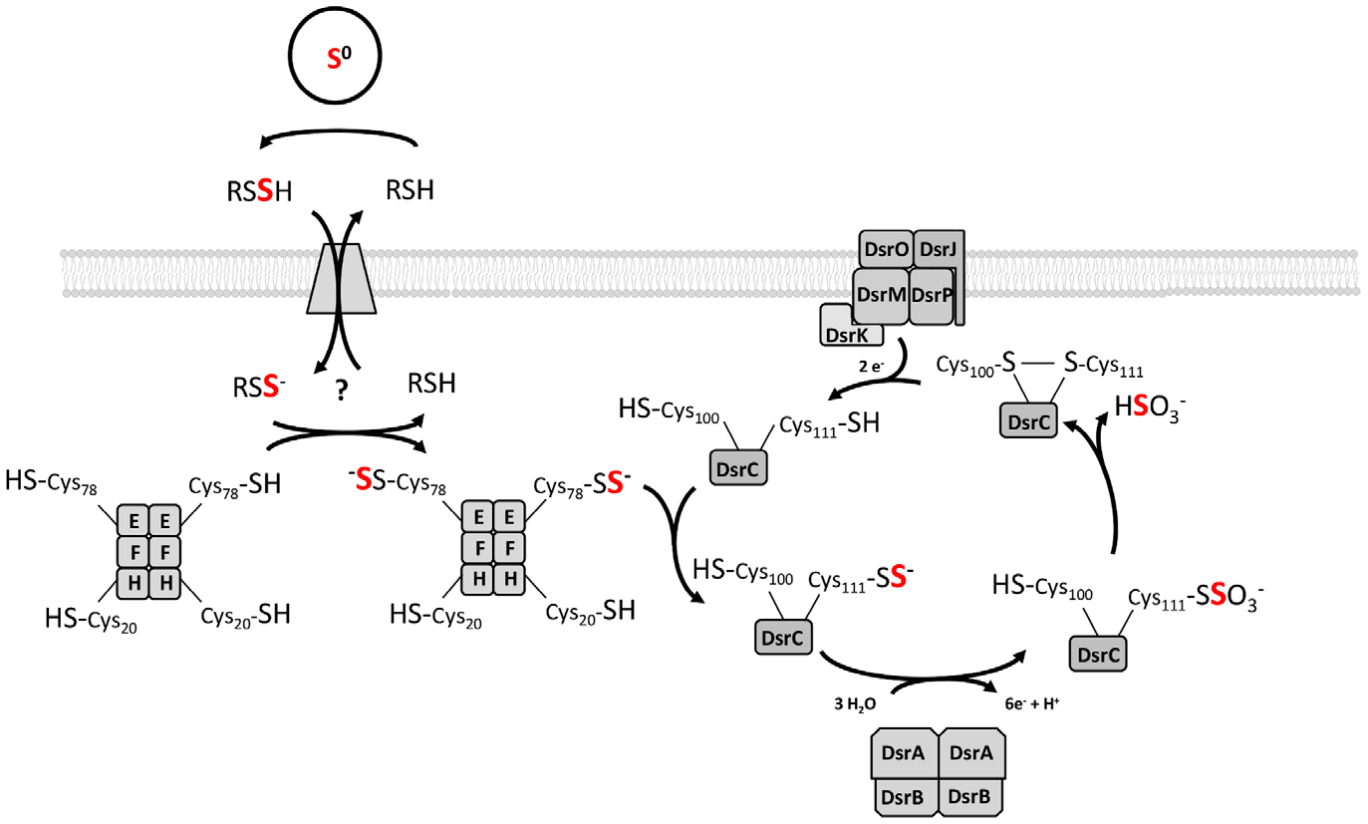
Model of sulfur oxidation in *Allochromatium vinosum* integrating a sulfur transfer function for DsrEFH and a substrate-donating function for DsrC. For better legibility, Dsr proteins are identified by capital letters only (DsrEFH: E, F, H). Thiol groups and persulfides are shown in the ionized or protonated state according to their supposed pK_a_ values of around 8.5 [41] and 6.2 [42], respectively. Since persulfides are 1 to 2 pK_a_ units more acidic than their thiol equivalents we calculated the pK_a_ of the assumed carrier molecule glutathione amide persulfide on the basis of the pK_a_ for glutathione [43] to be around 7.2. See discussion for detailed explanation.

The question whether the formation of the polysulfide chain bound to DsrC-Cys111 is also occurring *in vivo* remains unsolved. A polysulfide chain has also been found on the Sud protein from *Wolinella succinogenes*: a chain of up to ten sulfur atoms built up on this periplasmic sulfurtransferase under experimental conditions [24]. Another example for a protein binding polysulfide is the sulfide:quinone oxidoreductase from *Acidanus ambivales* in which a chain of three sulfur atoms bridges a pair of active site cysteine residues [25]. If the polysulfide chain we observed on DsrC indeed occurs *in vivo* sulfur atoms would have to be successively oxidized and released from DsrAB. In the structure of DsrAB from *Desulfovibrio vulgaris* Hildenborough [9] a channel for sulfite access to the active site was identified that is distinct from the large cavity that was originally proposed to serve as the access route for sulfite in DsrAB from the archaeal sulfate reducer *Archaeoglobus fulgidus* [26]. The large cavity described for the *A. fulgidus* sulfite reductase is almost completely occupied by the C-terminal arm of DsrC in the structures where DsrAB co-crystallized with DsrC [9], [10], [11] which was not the case for the *Archaeoglobus* protein [26]. The alternative, much narrower funnel proposed as entrance for sulfite by Oliveira et al. 2011 [9] is not blocked by DsrC binding and is also present in the other published DsrAB structures. If the same was true for *A. vinosum* DsrAB, sulfite molecules could in principle be successively released via this dedicated channel for sulfite. However, as explained above, sulfite is proposed to be released from sulfonated DsrC by formation of a disulfide bond between the two conserved cysteines of the protein’s carboxy-terminal arm (Fig. 7). A new catalytic cycle could then only start after reduction of this disulfide bond which would require release of disulfidic DsrC from DsrAB and interaction with the reducing protein. Taken together it seems more reasonable that the chain of three sulfur atoms bound to DsrC is an artifact and is caused by the lack of sulfur-converting DsrAB that is present *in vivo*.

Neither DsrEFH nor DsrC showed rhodanese or GSSH:cyanide sulfurtransferase activity. This lack of enzyme activity agrees with the hypothesis that a yet unknown sulfurtransferase transfers sulfur from the carrier molecule to DsrEFH. Instead of reacting directly with low molecular weight persulfides, DsrEFH and DsrC appear to act as shuttles that mediate the transfer of the sulfur from a cytoplasmic donor persulfide to DsrAB where it is further oxidized. This assumption is supported by a study on the regulation of *dsr* genes by Grimm et al. [27]. Compared to *dsrA* the transcription levels of *dsrEFH* and *dsrC* are significantly higher when *A. vinosum* is grown on sulfide. High copy numbers of DsrEFH and DsrC would guarantee sufficient substrate supply for DsrAB and a turnover rate that is high enough for efficient sulfur oxidation.

In summary, our results further support the following model for sulfur oxidation in *A. vinosum* (Fig. 7): DsrEFH serves as cytoplasmic acceptor for sulfur that is delivered by the carrier molecule for sulfur imported from the periplasm where the transiently stored sulfur globules are located. The transfer from the carrier molecule to DsrEFH is conducted by an unknown sulfurtransferase since DsrEFH cannot mobilize sulfane sulfur from low molecular weight thiols. Once the persulfide on DsrE-Cys78 is formed, the sulfur is relayed to DsrC-Cys111. DsrC finally passes on the sulfur to the reverse dissimilatory sulfite reductase DsrAB. After oxidation of the sulfane sulfur DsrC dissociates from the active site of DsrAB with a sulfonate group still bound to the protein. By formation of an intramolecular disulfide bond between DsrC-Cys100 and DsrC-Cys111 a sulfite molecule would be reductively released from DsrC. After the regeneration of the thiol groups DsrC can enter another cycle.

## Materials and Methods

### Strains and Media

The bacterial strains and plasmids used are described in Table S1. For molecular cloning *E. coli* DH5α was used. All *E. coli* strains were grown on LB medium [28]. *A. vinosum* DSM 180^T^ wild type and complementation mutants were cultivated as described previously [29]. *A. vinosum* strains were grown photoorganoheterotrophically on malate (RCV-medium [30]), using trace element solution SL12 [31]. For solidification of the medium 1% (w/v) phytagel Sigma-Aldrich, Munich, Germany) was added, as well as 0.5% NaCl to aid gelling and 0.02% (w/v) Na_2_S_2_O_3_× 5 H_2_O, 2 mM sodium acetate and 2.6 ml feeding solution (for 100 ml: 3.1 g NaSH× H_2_O, 5.0 g NaHCO_3_). For photolithoautotrophic growth of *A. vinosum* strains Pfennig’s medium [32] supplemented with 2 mM sulfide was used. Antibiotics were used at the following concentrations (in µg ml^−1^): for *E. coli*, kanamycin, 50; ampicillin, 100, for *A. vinosum*, kanamycin, 10; rifampicin, 50.

### Overproduction and Purification of Dsr Proteins

Wild type and mutated DsrEFH and DsrC proteins were overproduced and purified as described earlier [22], [8], [3]. All proteins carried an amino-terminal His-tag. In mutated proteins, the conserved cysteine residues were replaced by a serine residue.

### Preparation of Proteins for Electrophoretic Separation

The formation of DsrEFH/DsrC complexes was achieved by incubating 200 pmol DsrEFH and 400 pmol DsrC in 5 mM HEPES (pH 7.8), 0.1 M KCl, 0.01% Tween 20 and 25 µM TCEP in a final volume of 60 µl for 30 minutes at 30°C. The native gel was run at 4°C and 12 mA. After 60 minutes in Coomassie solution the gel was destained for 30 minutes. The stained gel pieces containing protein were cut out from the native gel and incubated in 50% acetonitrile for 10 minutes at RT and subsequently placed into the slots of a stacking gel for SDS-PAGE. The gel pieces were covered by a layer of 1 × Rotiload 1 (Roth, Karlsruhe, Germany) and incubated for 10 minutes before the run was started. The DsrEFH/DsrC complexes, prepared as above, were also analysed by Blue-native PAGE, as described in [33] without the use of α-aminocaproic acid, and run at 4°C and 6 mA.

For the electrophoretic separation of proteins by their molecular mass the protocol by Laemmli was used. For the separation of native proteins, the Laemmli method was modified by preparing buffers without SDS. The gels were stained with 0,25% Coomassie-Brilliant-Blue R250; 50% methanol; 10% acetic acid; destaining solution: 20% methanol; 10% acetic acid. In the case of Blue-native PAGE after Coomassie blue staining, the gel was subjected to silver staining.

### Dynamic Light Scattering (DLS)

DLS measurements were conducted using a Zetasizer Nano-ZS instrument (Malvern Instruments, Worcestershire, United Kingdom) equipped with a He-Ne (633 nm) laser light source. Samples were filtered through a 0.22 µm filter into a 3-mm path length quartz cell (Hellma, Mülheim, Germany) prior to analysis. Light scattering of DsrEFH (2 mg/ml) was measured in the presence or absence of DsrC (0.8 mg/ml), under the same ratio and conditions as described for native gels. Three measurement cycles were performed for each sample. Acquisition was performed at 30°C and the data were averaged from eight scans of 10 s each. Average protein diameter values and the distribution of sizes by volume were analyzed using the Malvern Instruments DTS software.

### Cloning, Overproduction and Purification of *E. coli* IscS

For the amplification of the *iscS* gene genomic DNA of *E. coli* K-12 served as template. *Nde*I and *Bam*HI restrictions sites were introduced via the primers *iscS*NdeIfor (5′-TATAGACATATGAAATTACCGATTTAT-3′) and *iscS*BamHIrev (5′-TGATTCCGAGGATCCTTAATGATGAGCCC-3′). After digestion the amplicon was ligated into the corresponding sites of pET15b (Novagen) resulting in plasmid pET15iscS. The overproduction of the amino-terminally His-tagged IscS was carried out in *E. coli* BL21(DE3). 500 ml LB medium containing ampicillin was inoculated with cells from 25 ml of an overnight culture and grown at 37°C and 180 rpm until an optical density of 0.6. Overproduction of the protein was induced with 1 mM IPTG. The cells were grown under the same conditions for another two hours. The protein was purified via Nickel-chelate affinity chromatography according to the manufactor’s instructions (QIAgen, Hilden, Germany).

### Construction of *A. vinosum* Δ*dsrE* Complementation Strains

The introduction of modified *dsrEFH* sequences under control of the *dsr* promoter into the *A. vinosum* Δ*dsrE* mutant strain was carried out according to [3]. Modified *dsrEFH* fragments were obtained from the respective expression plasmids pETEFH_20_, pETE_78_FH and pETE_78_FH_20_ [3] by digestion with *Nde*I and *Bam*HI. The fragments were subsequently ligated into the corresponding restrictions sites of pBBR1MCS2-L [12]. The resulting plasmids were transferred to *E. coli* S17-1 for conjugation with *A. vinosum* Δ*dsrE*. *E. coli* S17-1 cells and stationary phase cells of *A. vinosum*Δ*dsrE* were mixed 1∶3 and incubated anaerobically on cellulose nitrate membranes (pore size 0,45 µm; Sartorius, Göttingen) on solid RCV medium under constant illumination at 30°C according to the method described in [29]. After two days the cells were washed from the filter with 1 ml of RCV medium and applied to solid RCV medium containing kanamycin for the selection of transconjugants. Genotypes of the complemented strains were verified by colony PCR.

### Phenotypic Characterization of *A. vinosum in trans* Complementation Strains

Photolithoautotrophic growth of *Allochromatium vinosum* wild type and complementation mutants was performed as follows: 250 ml of photoorganoheterotrophically grown cultures were harvested (5900×g, 10 minutes, RT) and the resuspended cells were used as inoculum for 1 liter of modified Pfennig’s medium [34]. The cultures were kept at 30°C and constantly illuminated. To start the growth experiments, the cultures were supplemented with 2 mM NaSH × H_2_O and were observed until the wild type control had converted all sulfide to sulfate. Elemental sulfur was determined via cyanolysis [35], sulfate was measured by the method of Sörbo [36].

### Thiosulfate:cyanide Sulfurtransferase, Glutathione-persulfide:cyanide Sulfur-Transferase and Thiosulfate Reductase Activity

Thiosulfate:cyanide sulfurtransferase (rhodanese) activity was measured according to [37]. For the evaluation of thiosulfate reductase activity the method of Prieto et al. [38] was modified. 20 µM of each protein was incubated with 10 mM Tris-HCl, pH 8.5, 5 mM DTT and 10 mM Na_2_S_2_O_3_ in a final volume of 500 µl for 5 minutes at 20°C. The reaction was stopped with 500 µl of 0.23 M HgCl_2_ and centrifuged for 10 minutes at 14000 rpm. For quantification of sulfite generated by reduction of thiosulfate, 500 µl of the supernatant were incubated with 1 ml formaldehyde (0.02% v/v) and fuchsin (0.04% w/v in 0.72 M HCl) for 10 minutes at RT and finally measured at 600 nm.

To determine whether DsrEFH or DsrC can mobilize sulfur from GSSH the rhodanese assay by Ray et al. [37] was modified and GSSH was used as substrate instead of thiosulfate. GSSH as was formed by incubating 500 µM oxidized glutathione with 450 µM sulfide for 30 minutes at 30°C [39].

### Sulfur Binding and Transfer Experiments

For the sulfur binding experiments 30 µM of His-tagged protein was incubated either with 2 mM NaSH or with 2 µM IscS and 2 mM cysteine for one hour at 30°C in 100 µl 50 mM Tris-HCl, pH 7.5, containing 100 mM NaCl. Sulfite (2 mM), thiosulfate (2 mM) and GSSH (0.5 mM) were also tested as substrates.

For detecting sulfurtransferase activity the putative sulfur-donating protein was incubated with sulfide as described above. Afterwards, sulfide was removed by gel filtration on PD Mini–Trap columns (GE Healthcare, Munich, Germany). The columns were run according to the manufacturer’s instructions using a volume of 700 µl for elution. The successful removal of sulfide from the protein samples was verified by HPLC analysis [40]. For that purpose, samples of the first, second and third PD Mini–Trap elution steps were analyzed. The first eluate contained the persulfurated protein that was further used as sulfur donor, and was verified not to contain any sulfide. Free sulfide eluted in the second elution step. As an additional control, a sample containing sulfide but no protein was prepared. After the gel filtration, DsrC and DsrEFH were added to the first eluate, incubated for one hour at 30°C and analyzed via MALDI-TOF mass spectrometry. Residual sulfide in the first eluate would have been bound by the proteins. That was not the case as confirmed by mass spectrometric analyses of these control samples. A mass increase was not detected.

30 µM of the putative sulfur acceptor protein was added after the sulfide-free donor protein samples had been concentrated to their initial concentration of 30 µM using Vivaspin 500 centrifugal concentrators (5 kDa MWCO, Sigma-Aldrich, Munich, Germany). The samples were again incubated for one hour in a final volume of 100 µl containing 50 mM Tris-HCl, pH 7.5, and 50 mM NaCl. After buffer exchange the samples were stored overnight on ice.

### MALDI-TOF Mass Spectrometry

For MALDI-TOF mass spectrometry, the buffer was exchanged for 10 mM HEPES, pH 7.5 by using PD Mini–Trap columns (GE Healthcare, Munich, Germany). Samples were diluted 1∶5 with 0.1% trifluoracetic acid in MilliQ water and mixed with one volume of matrix: alpha-cyano-4-hydroxycinnamic and sinapic acid were used. Matrices were dissolved in 0.1% trifluoracetic acid in acetonitrile. Spectra were recorded in the linear positive mode within a range of 2 kDa to 20 kDa using a Biflex III (BrukerDaltonik GmbH, Leipzig, Germany).

### Surface Plasmon Resonance

A Biacore 2000 instrument coupled with a CM5 sensor chip (Biacore, GE Healthcare, Munich, Germany) was used for SPR measurements at 25°C using 10 mM HEPES, 150 mM NaCl, 3 mM EDTA, 0.005% Tween 20, pH 7.4, as running buffer. DsrEFH in 20 mM sodium acetate, pH 5.0, was covalently immobilized to the chip using the amine coupling protocol as recommended by the manufacturer. The protein (150 µg/ml) was injected during 2 minutes at 10 µl/min, resulting in ∼ 600 resonance units (RU) of immobilized protein on the CM5 chip surface. Flow cell 1 was similarly treated with buffer in the absence of the DsrEFH (control). The wild type and mutant DsrC proteins (10 µM) were first incubated in running buffer in the presence of 25 µM tris(2-carboxyethyl)phosphine (TCEP) for 30 minutes at RT. After reduction, DsrC was diluted and injected at flow rate of 40 µl/minute during 3 minutes. At the end of sample injection, the running buffer was flowed for 6 minutes over the sensor surface to allow dissociation, and then the surface was regenerated using 2 M MgCl_2_ for 30 s. All the sensorgrams were processed using the double referencing method to eliminate the nonspecific binding from background contribution and the buffer artifacts were removed by subtracting signals from the reference flow cell and from buffer blank injections. All binding experiments were run in duplicate.

## Supporting Information

Figure S1 **MALDI-TOF spectra of DsrC after incubation with sulfite.** 30 µM of DsrC proteins were incubated with 2 mM sulfite for 1 hour at 30°C. The binding of one molecule of sulfite is demonstrated by 80 Da mass increase.
(TIFF)

Table S1 Strains and plasmids used in this study.
(PDF)
