# Supplementary material for: Cytoplasmic Sulfurtransferases in the Purple Sulfur Bacterium Allochromatium vinosum: Evidence for Sulfur Transfer from DsrEFH to DsrC
Source: PLoS One. 2012 Jul 16;7(7):e40785. doi: 10.1371/journal.pone.0040785 (PMC3397948; doi:10.1371/journal.pone.0040785)
Supplement: Table S1 — Strains and plasmids used in this study. (PDF) [file pone.0040785.s002.pdf]

Table S 1. Bacterial strains and plasmids used in this study.

Strains and plasmids	Genotype and Phenotype	References or source
Strains		
Escherichia coli		
DH5α	F^{-} ϕ80d*lacZ*ΔM15 Δ(*lacZYA-argF*)*U169 recA1 endA1 hsdR17* (r_K^- m_K^+) *supE44*λ^- *thi-1 gyrA relA1*	[1]
BL21(DE3)	F^- ompT gal dcm lon hsdS_B(r_B^- m_B^-) λ(DE3 [lacI lacUV5-T7 gene 1 ind1 sam7 nin5])	Novagen
S17-1	294 (*recA pro res mod*$^+$) TprSmr (pRP4-2-Tc::Mu-Km::Tn7)	[2]
Allochromatium vinosum		
Rif50	Rif^r, spontaneous rifampin-resistant mutant of *A. vinosum* DSMZ 180^T	[3]
Δ*dsrE*	Rif^r, Rif50 with *in-frame* deletion of *dsrE*	[4]
Δ*dsrE* + *dsrE78FH*	Rif^r, Kan^r, deletion mutant Δ*dsrE* carrying the plasmid pBBR1MCS2*dsr*E_{78}*FH*	this work
Δ*dsrE* + *dsrEFH20*	Rif^r, Kan^r, deletion mutant Δ*dsrE* carrying the plasmid pBBR1MCS2*dsrEF*H_{20}	this work
Δ*dsrE* + *dsrE78FH20*	Rif^r, Kan^r, deletion mutant Δ*dsrE* carrying the plasmid pBBR1MCS2*dsr*E_{78}*F*H_{20}	this work
Plasmids		
pETEFH	Ap^r, T7 promotor, His-Tag, *Nde*I/*Bam*HI fragment of amplified *dsrEFH*	[5]
pETE_{78}FH	Ap^r, T7 promotor, His-Tag, *Nde*I/*Bam*HI fragment of amplified *dsr*E_{78}*FH*	[4]
pETEFH_{20}	Ap^r, T7 promotor, His-Tag, *Nde*I/*Bam*HI fragment of amplified *dsrEF*H_{20}	[4]
pETE_{78}FH_{20}	Ap^r, T7 promotor, His-Tag, *Nde*I/*Bam*HI fragment of amplified *dsr*E_{78}*F*H_{20}	[4]
pETCEX	Ap^r, T7 promotor, His-Tag, *Nde*I/*Bam*HI fragment of amplified *dsrC*	[6]
pETCEXSer100	Ap^r, T7 promotor, His-Tag, *Nde*I/*Bam*HI fragment of amplified *dsr*C_{100}	[6]
pETCEXSer111	Ap^r, T7 promotor, His-Tag, *Nde*I/*Bam*HI fragment of amplified *dsr*C_{111}	[6]
pETCEXSer100/111	Ap^r, T7 promotor, His-Tag, *Nde*I/*Bam*HI fragment of amplified *dsr*$C_{100/111}$	[6]
pET15*iscS*	Ap^r, T7 promotor, His-Tag, *Nde*I/*Bam*HI fragment of amplified *iscS*	this work
pBBRMCS2-L	Kan^r, *dsr* promotor, *Nde*I/*Bam*HI fragment of amplified *dsrL*	[3]
pBBRMCS2*dsrEFH*	Kan^r, *dsr* promotor, *Nde*I/*Bam*HI fragment of amplified *dsrEFH*	[4]
pBBR1MCS2*dsr*E_{78}*FH*	Kan^r, *dsr* promotor, *Nde*I/*Bam*HI fragment of *dsr*E_{78}*FH*	this work
pBBR1MCS2*dsrEF*H_{20}	Kan^r, *dsr* promotor, *Nde*I/*Bam*HI fragment of *dsrEF*H_{20}	this work
pBBR1MCS2*dsr*E_{78}*F*H_{20}	Kan^r, *dsr* promotor, *Nde*I/*Bam*HI fragment of *dsr*E_{78}*F*H_{20}	this work

References

1. Hanahan D (1983) Studies on transformation of *Escherichia coli* with plasmids. J Mol Biol 166: 557-580.
2. Simon R, Priefer U, Pühler A (1983) A broad host range mobilization system for *in vivo* genetic engineering: transposon mutagenesis in gram negative bacteria. Bio/Technology 1: 784-791.
3. Lübbe YJ, Youn H-S, Timkovich R, Dahl C (2006) Siro(haem)amide in *Allochromatium vinosum* and relevance of DsrL and DsrN, a homolog of cobyrinic acid *a,c* diamide synthase for sulphur oxidation. FEMS Microbiol Lett 261: 194-202.
4. Dahl C et al. (2008) Structural and molecular genetic insight into a wide-spread bacterial sulfur oxidation pathway. J Mol Biol 384: 1287-1300.
5. Dahl C, Schulte A, Shin DH (2007) Cloning, expression, purification, crystallization and preliminary X-ray diffraction analysis of DsrEFH from *Allochromatium vinosum*. Acta Cryst F63: 890-892.
6. Cort JR et al. (2008) *Allochromatium vinosum* DsrC: solution-state NMR structure, redox properties and interaction with DsrEFH, a protein essential for purple sulfur bacterial sulfur oxidation. J Mol Biol 382: 692-707.
